# Full-Length Enrich c-DNA Libraries-Clear Cell-Renal Cell Carcinoma

**DOI:** 10.1155/2012/680796

**Published:** 2012-03-20

**Authors:** Sai-Wen Tang, Jung-Yaw Lin

**Affiliations:** Institute of Biochemistry and Molecular Biology, College of Medicine, National Taiwan University, No. 1 Jen-Ai Road First Section, Taipei 100, Taiwan

## Abstract

Clear cell renal cell carcinoma (ccRCC), the most common subtype of RCC, is characterized by high metastasis potential and strong resistance to traditional therapies, resulting in a poor five-year survival rate of patients. Several therapies targeted to VEGF pathway for advanced RCC have been developed, however, it still needs to discover new therapeutic targets for treating RCC. Genome-wide gene expression analyses have been broadly used to identify unknown molecular mechanisms of cancer progression. Recently, we applied the oligo-capping method to construct the full-length cDNA libraries of ccRCC and adjacent normal kidney, and analyzed the gene expression profiles by high-throughput sequencing. This paper presents a review for recent findings on therapeutic potential of MYC pathway and nicotinamide N-methyltransferase for the treatment of RCC.

## 1. Introduction

Renal cell carcinoma (RCC) represents 3% of all human malignancies worldwide with an increasing incidence and accounts for 85% of renal cancers, resulting in close to 78,000 deaths annually [1–5]. The most common subtype, clear cell RCC (ccRCC), which originates from the proximal tubule epithelium, is mostly sporadic, unilateral, and unifocal [6]. RCC cells have a poor response to traditional therapies, such as the chemotherapy, hormonal therapy, and radiation therapy [7–10]. These properties result in a poor prognosis and low five-year survival rate of RCC patients. 

Surgery remains to be the main therapeutics for treatment of RCC [11]. It has been demonstrated that the activation of hypoxia-inducible factor-*α* (HIF-*α*) turns on the genes, such as VEGF and PDGF, which are responsible for the progression of ccRCC, providing potential targets for advanced ccRCC [12].

Gene expression analysis appears to be an important tool for studying cancer pathogenesis and progression. Markers related to tumor proliferation, growth, angiogenesis, and loss of cell adhesion have been evaluated for their potential as prognostic factors. Numerous reports have investigated the differential gene expression profile between normal and tumor tissues using the high-throughput technologies, including cDNA microarray, cDNA subtraction, and serial analysis of gene expression [13–15]. Many studies have applied these methods to analyze the genome-wide changes in ccRCC, providing specific gene expression signature, the potential biomarkers, and prognostic factors [16–21], whereas Lenburg et al. pointed out a diverse gene expression results by microarray method published previously [22]. Recently, two international projects, The Cancer Genome Atlas (TCGA) and International Cancer Genome Consortium (ICGC), have been organized to provide the platform for the integration and comparison of cancer genomic abnormalities identified by independent research groups [23–25].

The oligo-capping method developed by Dr. Suzuki and Dr. Sugano at the Institute of Medical Science of Tokyo University was applied to construct the full-length enriched cDNA library for the analysis of transcriptional start sites of genes [26, 27]. Yamada et al. utilized the oligo-capping method to explore gene expression profiles of hepatoblastomas and the corresponding normal livers and identify a poor-prognostic indicator for hepatoblastomas [28]. To investigate the differentially expressed genes in ccRCC, the full-length enriched cDNA libraries of ccRCC and normal kidney tissues were constructed and sequenced. We identified 383 differentially expressed genes, and further confirmed the differential expression of 20 ccRCC-associated genes, which have not been reported to be dysregulated in ccRCC. By functional network analysis using 383 differentially expressed genes, MYC pathway was identified to be activated in neoplastic disease [29]. By using MYC siRNA, the importance of MYC was further demonstrated in ccRCC cells.

Additionally, nicotinamide N-methyltransferase (NNMT) was identified as the ninth of 201 upregulated genes with 65.2-fold increase in full-length cDNA-enriched libraries of ccRCC tissues. We showed that NNMT contributes to MMP-2 expression to induce cell invasiveness via PI3K/AKT/SP1 pathway, suggesting NNMT as a novel invasive-related gene for ccRCC. Our results elucidate the novel functions of NNMT in ccRCC and suggest that NNMT could be considered as a potential therapeutic target of ccRCC.

## 2. Gene Expression Profiles of ccRCC and Normal Kidney

To construct the full-length enriched cDNA libraries from two tissues of ccRCC (Grade II/Stage I; Grade II/Stage II) and adjacent normal kidney, the oligo-capping method developed by Y. Suzuki and S. Sugano was performed. We successfully sequenced 19,425 and 12,400 colonies of ccRCC and normal kidney cDNA libraries, respectively. The sequencing results were blasted to the UniGene database of NCBI using the BLAST program. The blast result with the score more than 200 was denoted as a gene, and a total of 4,356 and 3,055 genes were identified in the full-length enriched cDNA libraries from ccRCC and normal kidney tissues, respectively. The gene expression patterns of ccRCC and normal kidney were further compared to identify differentially expressed genes. By the definition that the genes with the colony number in ccRCC three-fold higher or lower than that in normal kidney were considered as the ccRCC-associated genes, 201-upregulated and 182-downregulated genes were identified [30].

## 3. Classification of ccRCC-Associated Genes by Cellular Function

The differentially expressed genes in ccRCC were classified into groups by their cellular functions according to gene ontology (GO) term in NCBI [30]. We observed that many metabolism-related genes were differentially expressed in ccRCC, such as NNMT, lysyl oxidase, transglutaminase 2, heme oxygenase 1, argininosuccinate synthetase, alcohol dehydrogenase 6, and phosphoenolpyruvate carboxykinase 1, indicating that the ccRCC cells might lose their normal functions as the kidney cells, and the metabolism-related genes might play a role in cancer progression. Additionally, the differential expression of several signal transduction-related genes was found, including secreted frizzled-related protein 1, insulin-like growth factor-binding protein 3, regulator of G-protein signaling 1, and frizzled-related protein, implying the activation of signaling pathways associated with the process of carcinogenesis. The genes involved in cellular proliferation were also found to be dysregulated. For instance, transforming growth factor (beta-induced), CD74 antigen, alpha-2-glycoprotein 1, inhibin, cyclin D1, and cell division cycle 42 were differentially expressed. The results might support the high proliferation ability of ccRCC cells. Interestingly, we also observed the altered expression of genes which function as cytoskeleton assembly, protease or protease inhibitor, such as gelsolin, MMP-2, cathepsin S, kininogen 1, alpha-1 antiproteinase member 5 and tissue inhibitor of metalloproteinase 3, providing the strong invasive activity of ccRCC cells.

## 4. Differential Expression of the Novel ccRCC-Associated Genes in ccRCC

The differential expression of the ccRCC-associated genes, which have not been reported to be dysregulated in ccRCC, was further studied in ccRCC tissue pairs by Q-PCR analysis (Table 1). Interestingly, the expression of the modulators of the Wnt signaling pathway, secreted frizzled-related protein 2 and 4, were significantly upregulated 34.9-, and 4.3-fold, respectively. In addition, the detoxification-related gene, glutathione S-transferase A3 was found to be significantly downregulated in ccRCC, indicating an elevated oxidative stress in ccRCC tissues. The overexpression of secreted protein acid rich in cysteine (SPARC) has been demonstrated in various cancers. Previous reports have shown that SPARC is involved in tumor development [31, 32], and in this study we also observed the upregulation of SPARC expression in ccRCC, suggesting the important role of SPARC in ccRCC (Table 1).

## 5. Activation of MYC Pathway in ccRCC

To identify the deregulated cellular pathways in ccRCC, functional network analysis was performed with the 383 ccRCC-associated genes by using Ingenuity Pathway Analysis, which has been used to identify significant pathways related to tumorigenesis in several tumors [33, 34]. The list of the ccRCC-associated genes was uploaded to the Ingenuity system and used as focus genes to produce the biological networks. The *P* values were calculated to represent the statistical significance that the focus genes in a network are randomly found together. The results of Ingenuity Pathway Analysis revealed that 17 networks were statistically significant and contained more than 10 ccRCC-associated genes. The top-ranking biological functions of each network were assigned according to Ingenuity Pathways Knowledge Base. The five biological networks with the highest score were found to be functionally related to cancer progression, such as cell cycle, cellular growth, or cellular movement (Table 2). 

**Figure 1:**
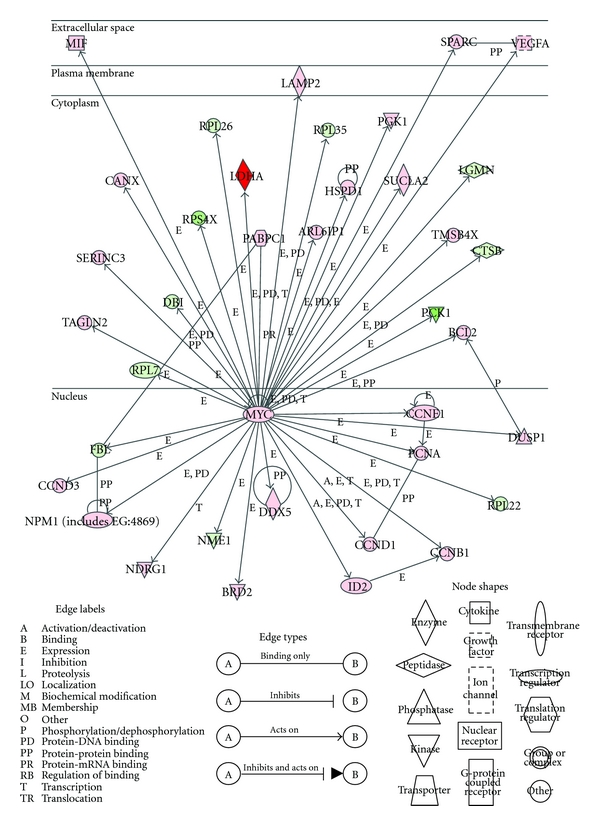
MYC and 37 MYC-target genes in ccRCC. Nodes represent genes, and their shapes indicate their functional categories. The colors of the nodes show the fold changes of the differentially expressed genes between ccRCC and normal kidney tissues (red, upregulated genes; green, downregulated genes). Edge labels and shapes represent the biological relationships between two nodes.

Interestingly, the top functional network with the highest score was composed of 35 differentially expressed genes and was assembled around the oncogene, MYC, with a set of MYC-target genes. The crucial role of MYC has been demonstrated in numerous kinds of human tumors and the regulation of cancer-related gene expression [35]. The upregulation or amplification of MYC in ccRCC has been previously reported [36–39]. Recent reports showed that HIF-2*α* promotes the transcriptional activity of MYC, whereas HIF-1 suppresses it [40, 41]. We further identified the MYC transcriptional targets among the 383 differentially expressed genes in ccRCC by using Ingenuity Pathways Knowledge Base. The differentially expressed genes with the connection to MYC labeled as expression (E), transcription (T), or protein-DNA interaction (PD) were considered as the MYC-target genes. As shown in Figure 1, 37 differentially expressed genes were found to be the MYC-target genes, suggesting that the MYC pathway members may be activated in ccRCC tissues, indicating that MYC pathway is activated and plays an important role in carcinogenesis of ccRCC. 

Furthermore, the upregulated expression of MYC was examined in 44 ccRCC tissue pairs by Q-PCR analysis. The average expression level of MYC in ccRCC tissues was significantly higher than that in normal kidney tissues. The results of immunohistochemical staining analysis also demonstrated that 24 (96%) of 25 ccRCC tissues showed the positive staining for MYC, including 15 cases with 3+ staining, 7 cases with 2+ staining, and 2 cases with 1+ staining, but negative staining for nonneoplastic renal tubules. The expression levels of MYC were also examined in 786O, 769P, A498, ACHN, and Caki-1 cells, which were derived from patients with ccRCC, and the results revealed that all of these ccRCC cell lines displayed a significant upregulation of MYC as compared with that of a pool of 5 normal tissues. These results suggest that the expression of MYC is markedly increased in ccRCC tissues and cell lines [30]. 

To investigate whether the expression of MYC pathway signature is enhanced due to MYC activation, the expression levels of the MYC-target genes, BCL2, CCND1, PCNA, PGK1, and VEGFA, which have been reported to be functionally related to cancer progression [42, 43], were further investigated in 44 ccRCC tissues by Q-PCR analysis. As shown in Table 3, the significant upregulation of these MYC pathway members was demonstrated. By analysis of the Pearson correlation method, we observed that the expression levels of BCL2, CCND1, PCNA, PGK1, and VEGFA were correlated with the levels of MYC in the tumor tissues of ccRCC (Table 3). These results indicate that the MYC pathway signature is activated and is associated with MYC upregulation in ccRCC tissues. 

## 6. Suppressing the Expression of MYC-Target Genes by MYC siRNA

We have demonstrated the correlation between the expression levels of MYC and the MYC-target genes in ccRCC tissues (Table 3). Therefore, whether the expression of the MYC-target genes is suppressed by the knockdown of MYC expression in ccRCC cells were investigated. The results of Q-PCR analysis revealed that treatment of MYC-specific siRNA significantly reduced expression levels of MYC-target genes BCL2, CCND1, PGK1, PCNA, and VEGFA in 769P cells [30]. It has been demonstrated that MYC is able to bind to specific DNA sequences (E-box motif, CACGTG) to function as a transcription factor [31]. Therefore, whether MYC could be directly recruited to the promoter regions of these MYC-target genes was studied. The potential E-box sites on the promoters of BCL2, CCND1, and PCNA were identified by blasting the sequence, CACGTG, with the potential promoter regions of BCL2, CCND1, and PCNA, and the primer pairs to specifically amplify the promoter regions containing the potential E-box sites were generated using Beacon Designer 4 program. The primers to amplify the E-box sites in the promoters of PGK1 and VEGFA have been described in previous studies. The Chromatin immunoprecipitation (ChIP) assay was carried out using these primers to amplify the potential MYC-binding regions in BCL2, CCND1, PCNA, PGK1, and VEGFA promoters. The endogenous MYC was observed to bind onto the promoter regions of BCL2, CCND1, PCNA, PGK1, and VEGFA [30]. These results indicate that MYC expression is able to enhance the expression levels of MYC-target genes by binding to their promoters in ccRCC cells. 

Previous studies have demonstrated that MYC plays a role in cellular transformation [29]. To address whether the expression of MYC is required for the malignant ability of ccRCC cells, the effects of the knockdown of MYC expression by RNAi technique was investigated in ccRCC cell lines, 769P and Caki-1 cells. As displayed in Figure 4(a), the expression level of MYC in 769P and Caki-1 cells were markedly suppressed by MYC-specific siRNA in a dose-dependent manner. The results of MTT assay revealed that the proliferation rate of 769P and Caki-1 cells was significantly reduced by knockdown of MYC expression in a dose-dependent manner (Figure 4(b)). Additionally, treatment with 20 nM of MYC-specific siRNA dramatically suppressed 95% and 91% of anchorage-independent growth ability in 769P cells and Caki-1 cells as shown by soft agar assay, respectively [30]. These results indicate that RNAi-mediated knockdown of MYC strongly suppresses the proliferation and anchorage-independent growth of ccRCC cells. 

It has been shown that MYC promotes cell cycle progression of cancer cells to enhance an uncontrolled cellular proliferation in various malignances [43]. Therefore, we further investigate whether the knockdown of MYC expression has the capacity of arresting cell cycle progression in ccRCC cells. Cell cycle distribution of 769P cells was analyzed by PI staining and flow cytometry analysis after treated with MYC siRNA. The results showed a significant increase of G0/G1 phase population of MYC siRNA-transfected cells as compared with the control cells. Additionally, the population in the process of DNA synthesis (S phase) was markedly decreased in 769P cells treated with MYC-specific siRNA [30]. These results show that a reduction of MYC expression disrupted the G1/S phase transition of 769P cells.

## 7. Overexpression of NNMT in ccRCC Tissues

**Figure 2:**
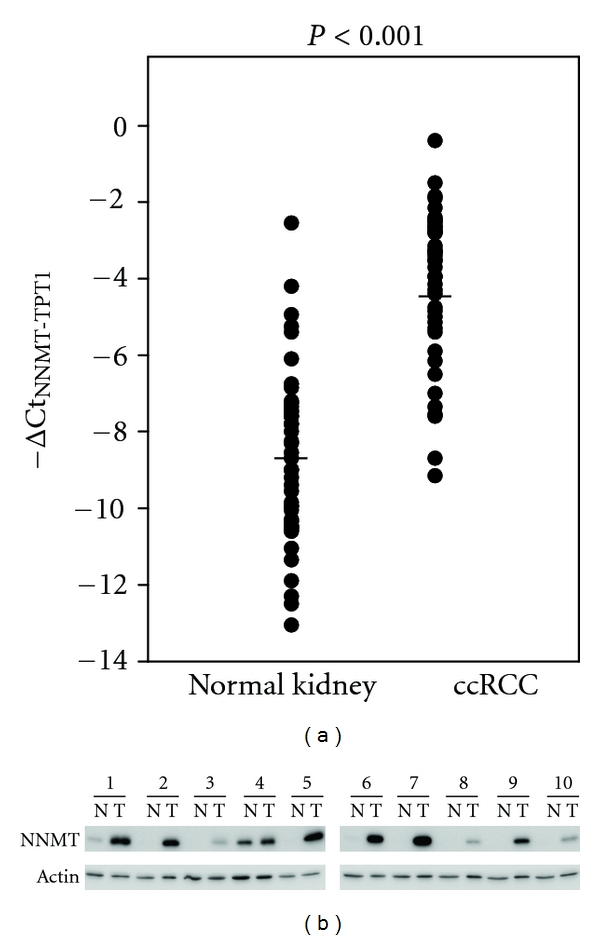
Overexpression of NNMT in ccRCC tissues. (a) mRNA expression levels of NNMT of 33 tissue pairs of ccRCC measured by Q-PCR analysis. Expression levels are represented by −ΔCt_NNMT-TPT1_ values. Each dot represents one tissue sample, and horizontal bars show mean values. *P* values are determined using a paired-samples Student's *t* test. (b) Western blot analysis to determine expression levels of NNMT protein in 10 ccRCC tissue pairs. N and T represent lysates from normal and tumor parts, respectively. Actin was used as a loading control.

Among these differentially expressed genes identified by analysis of full-length cDNA libraries in ccRCC, NNMT was one of the most upregulated genes with 65.2-fold overexpression. Previous studies have demonstrated that NNMT is overexpressed in ccRCC tissues and has the potential to be a ccRCC biomarker [44, 45]. To investigate whether NNMT is overexpressed in ccRCC tissue pairs, Q-PCR analysis was used to examine the mRNA levels of NNMT in these tissue pairs. The results showed that the expression of NNMT in 27 of 33 ccRCC tumors was highly upregulated (T/N > 3.0) by an average of 52.8-fold compared with that in adjacent normal kidney tissues (Figure 2(a)). The results of Western blot analysis also demonstrated a marked increase in NNMT protein levels in ccRCC tissues (Figure 2(b)). These results suggest that NNMT is overexpressed in ccRCC, and it was also reported that the overexpression of NNMT was found in colorectal cancer, papilloma thyroid cancer, and gastric cancer [46–48]. 

## 8. Identification of the Potential Role of NNMT in ccRCC

**Figure 3:**
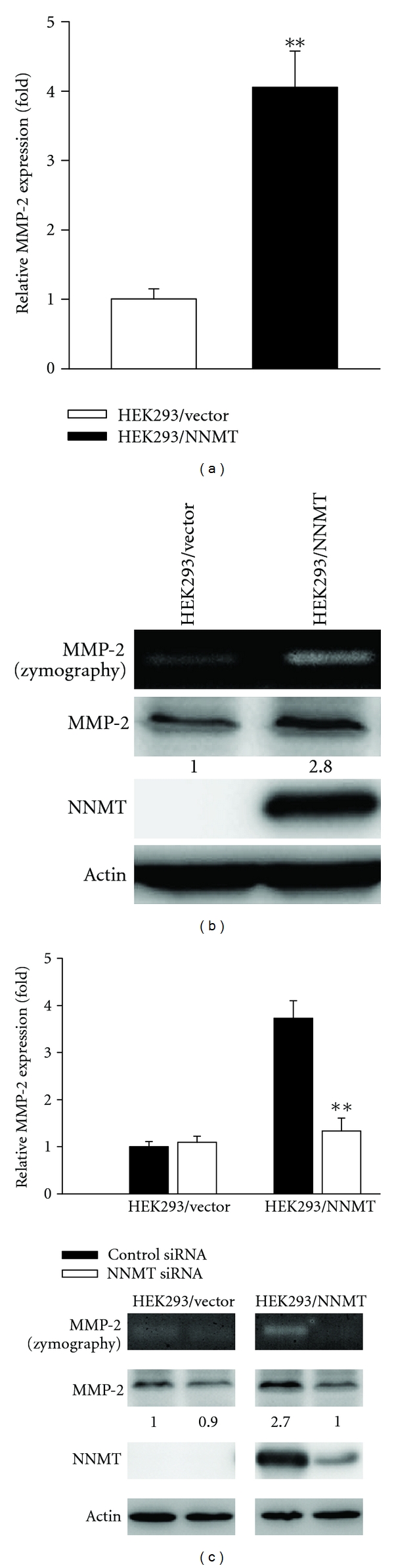
Effects of NNMT overexpression on the expression of MMP-2. (a) The expression levels of MMP-2 mRNA in HEK293/vector (control) and HEK293/NNMT cells were determined by Q-PCR and normalized to GAPDH. Data are presented as the mean of triplicate replications. S.D. is indicated by error bars. **P < 0.001  versus control cells. (b) Gelatin zymography assay was used to examine the MMP-2 activity in conditioned media, and western blot analysis to detect MMP-2 and NNMT in cell lysates from HEK293 cells with or without NNMT expression. (c) HEK293/vector and HEK293/NNMT cells were transfected with 40 *μ*mol/L of NNMT siRNA or control siRNA. Q-PCR (upper) gelatin zymography and Western blot analyses (lower) were performed to determine the expression levels of NNMT and MMP-2. Q-PCR data are presented as the mean of triplicate replications. S.D. is indicated by error bars. **P < 0.001 versus control cells. Actin was used as loading control.

Interestingly, Wu et al. recently demonstrated that a correlation between NNMT and cancer cell migration has been reported in bladder cancer [49]. We further investigated the expression levels of genes related to cell invasiveness in HEK293/NNMT cells using Q-PCR analysis, and MMP-2 expression was found to be enhanced by NNMT overexpression. As shown in Figure 3(a), MMP-2 expression of HEK293/NNMT cells was enhanced 4-fold compared with that of HEK293/vector cells. The results of gelatin zymography and western blot analyses demonstrated a significant increase of MMP-2 protein level in HEK293/NNMT cells (Figure 3(b)). Moreover, by treating siRNA specific to NNMT gene, the expression of NNMT was significantly suppressed and was accompanied by a reduction of MMP-2 expression in HEK293/NNMT cells (Figure 3(c)). 

**Figure 4:**
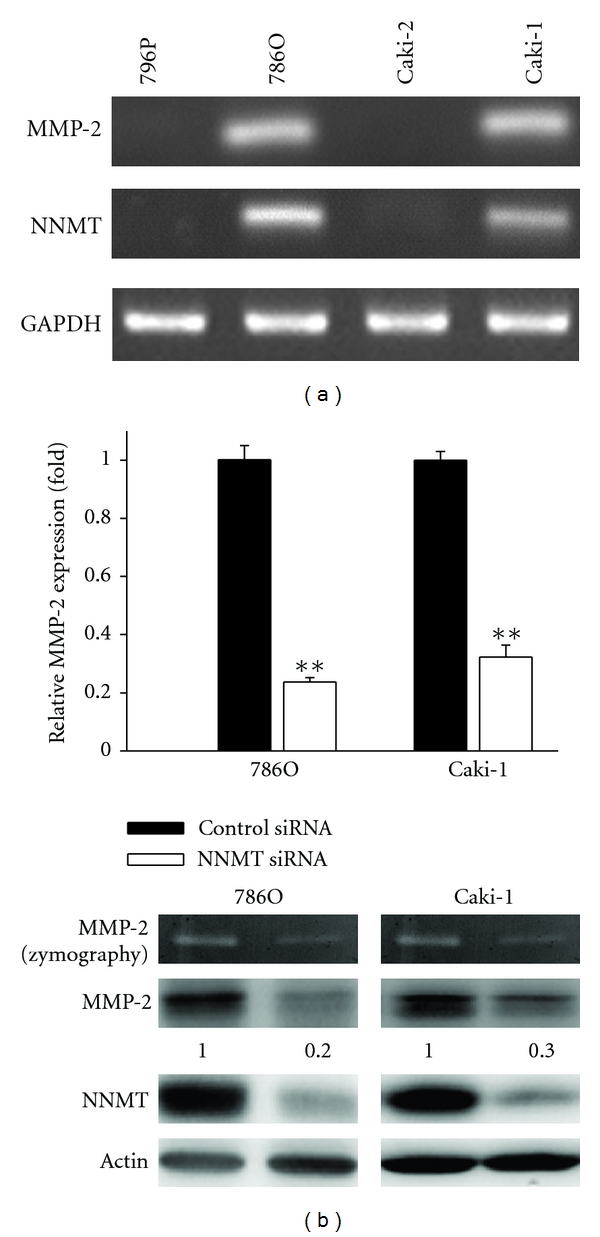
Effects of NNMT siRNA on MMP-2 expression in ccRCC cells. (a) Expression levels of MMP-2 and NNMT in ccRCC cell lines. Q-PCR was performed to determine MMP-2 and NNMT expression in ccRCC cell lines, 796P, 786O, Caki-2, and Caki-1. GAPDH was used as an internal control. (b) 786O and Caki-1 cells were transfected with 40 *μ*mol/L of NNMT siRNA or control siRNA. Q-PCR (upper), gelatin zymography and western blot analyses (lower) were performed to determine the expression levels of NNMT and MMP-2. Q-PCR data are presented as the mean of triplicate replications. S.D. is indicated by error bars. **P < 0.001 versus control cells. Actin was used as a loading control.

The effects of silenced NNMT expression on the MMP-2 expression were further investigated in ccRCC cell lines. Figure 4(a) indicated that 786O and Caki-1 cells (invasive ccRCC cell lines) had a higher NNMT and MMP-2 expression than those of 769P and Caki-2 cells (primary ccRCC cell lines). The results of siRNA treatment showed that the knockdown of NNMT expression resulted in the suppression of MMP-2 expression in 786O and Caki-1 cells (Figure 4(b)). These results suggest that NNMT expression is involved in MMP-2 expression. 

**Figure 5:**
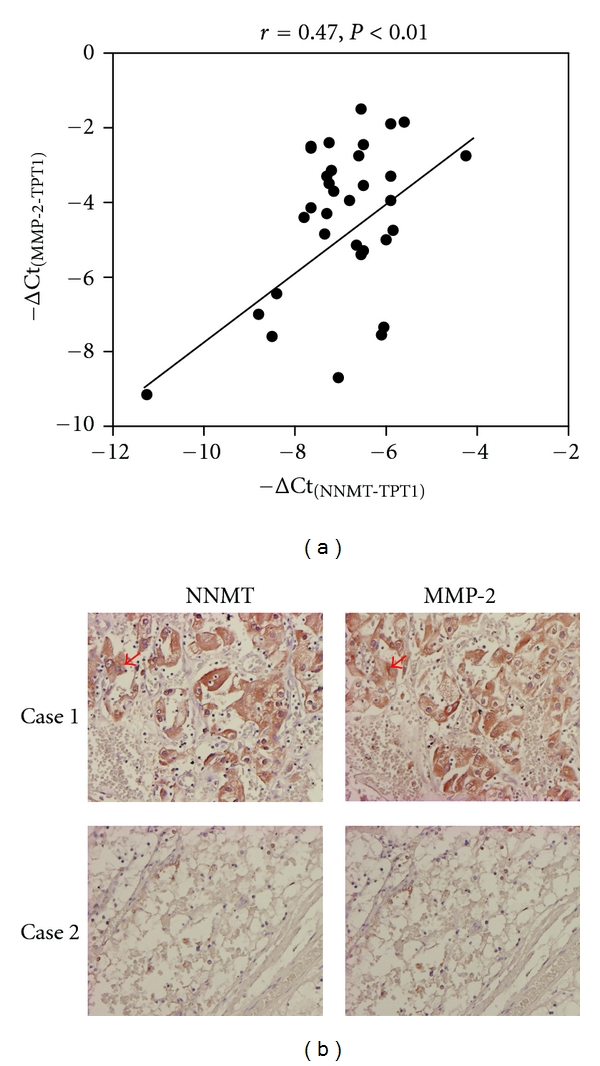
Correlation between the expression levels of NNMT and MMP-2 in ccRCC tissues. (a) Expression levels of NNMT and MMP-2 in 33 ccRCC tissues were dotted by using the −ΔCt values, and the correlation between the expression of NNMT and MMP-2 was analyzed by the *Pearson* correlation method (*r* = 0.47, *P* < 0.01). (b) Immunohistochemistry analysis was used to detect NNMT and MMP-2 in ccRCC tissue sections. The representative cases with a positive (case 1) or negative (case 2) immunostaining of NNMT and MMP-2 are shown. Arrow indicates the cytoplasm immunostaining of NNMT and MMP-2. Photos were taken under 100x magnification.

Moreover, we examined whether the expression of NNMT and MMP-2 was correlated in ccRCC tissues, and the results indicated a positive correlation between the mRNA levels of NNMT and MMP-2 in ccRCC tissues based on the statistical analysis using the *Pearson* correlation method (*r* = 0.47). The results of immunohistochemistry analysis also demonstrated that NNMT was overexpressed in 76% of ccRCC tissues, whereas the expression of MMP-2 was upregulated in 58% of ccRCC tissues. By the analysis of the *Pearson*'s chi-square test, the expression of NNMT and MMP-2 was observed to be significantly correlated in ccRCC tissues (Figure 5(a)), and the representative cases were shown in Figure 5(b). These results indicate that the expression of NNMT and MMP-2 is correlated in ccRCC tissues.

## 9. PI3K/AKT/SP1 Pathway Is Involved in NNMT-Mediated MMP-2 Expression

**Figure 6:**
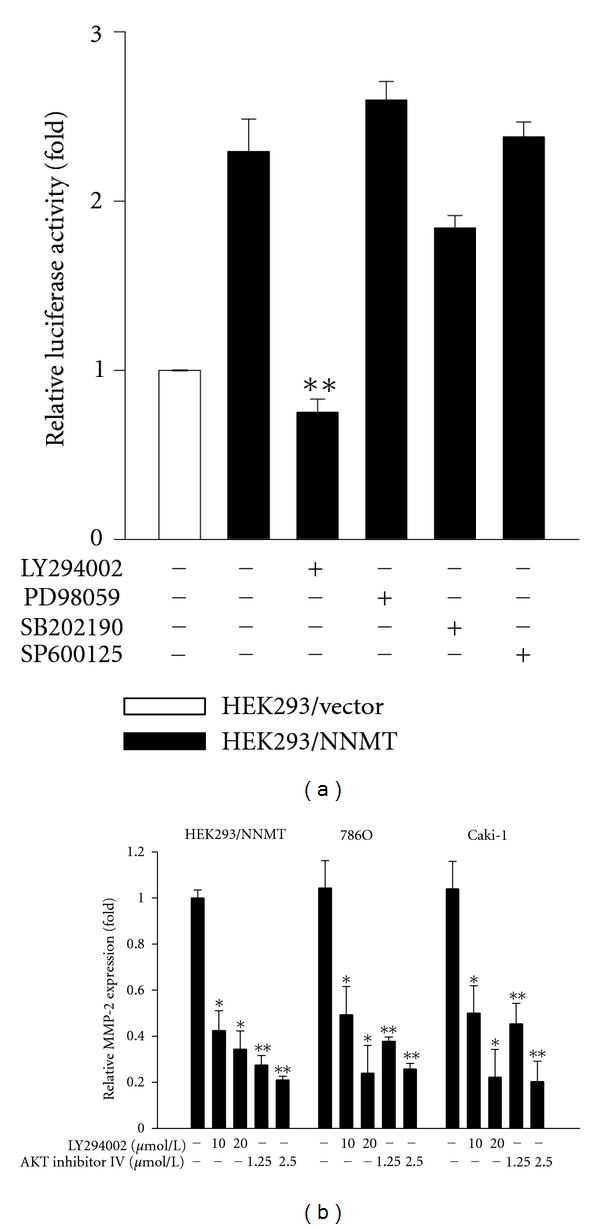
Signaling pathway involved in NNMT-mediated MMP-2 expression. (a) Various signaling pathway inhibitors were used to treat HEK293/NNMT cells transfected with full-length MMP-2 promoter plasmid and control reporter plasmid. The MMP-2 promoter activity was measured by luciferase reporter assay. Data are presented as the mean of triplicate replications. S.D. is indicated by error bars. **P < 0.001 versus untreated HEK293/NNMT cells. (b) HEK293/NNMT, 786O and Caki-1 cells were treated with LY294002 or AKT inhibitor IV for 18 h, and the expression of MMP-2 was measured by Q-PCR. Data are presented as the mean of triplicate replications. S.D. is indicated by error bars. *P < 0.05; **P < 0.001 versus untreated cells.

To identify which signaling pathway may participate in MMP-2 expression induced by NNMT, the signaling-pathway inhibitors, HEK293/NNMT cells were treated with the signaling-pathway inhibitors LY294002 (PI3K), PD98059 (ERK1/2), SB203580 (p38), and SP600125 (JNK), and then luciferase reporter assay was performed to measure the transcriptional activity of the full-length MMP-2 promoter. The results showed that treating with LY294002, but not SB203580, PD98059, and SP600125, markedly suppressed MMP-2 promoter activity in HEK293/NNMT cells (Figure 6(a)). Additionally, the expression levels of MMP-2 in HEK293/NNMT, 786O, and Caki-1 cells were significantly reduced by the treatment of LY294002 and AKT inhibitor IV (Figure 6(b)), indicating the importance of PI3K/AKT pathway on NNMT-mediated MMP-2 expression. 

**Figure 7:**
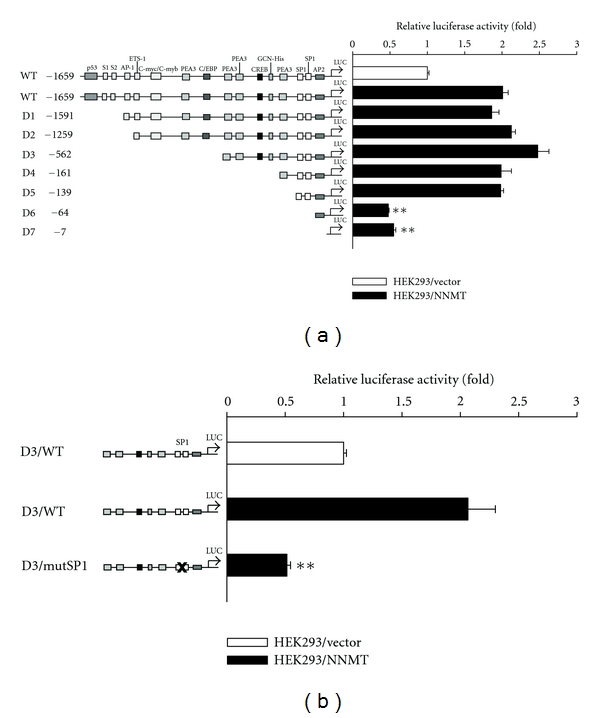
Role of SP1-binding elements in MMP-2 transcriptional activity. (a) The diagrams of the reporter constructs of full-length or various deletion mutants of MMP-2 promoter (D1–D7) are depicted on the *left*. The solid lines are the regions cloned upstream of the luciferase gene in the pGL3 luciferase reporter vector. The luciferase activity of cell extracts was analyzed by luciferase reporter assay. Data are presented as the mean of triplicate replications. S.D. is indicated by error bars. **P < 0.001 versus HEK293/NNMT cells transfected with full-length MMP-2 promoter. (b) Effects of site-specific mutation of the two putative SP1-binding sites on MMP-2 promoter activity were examined by luciferase reporter assay. Data are presented as the mean of triplicate replications. S.D. is indicated by error bars. **P < 0.001 versus HEK293/NNMT cells transfected with wild-type D3 construct.

**Figure 8:**
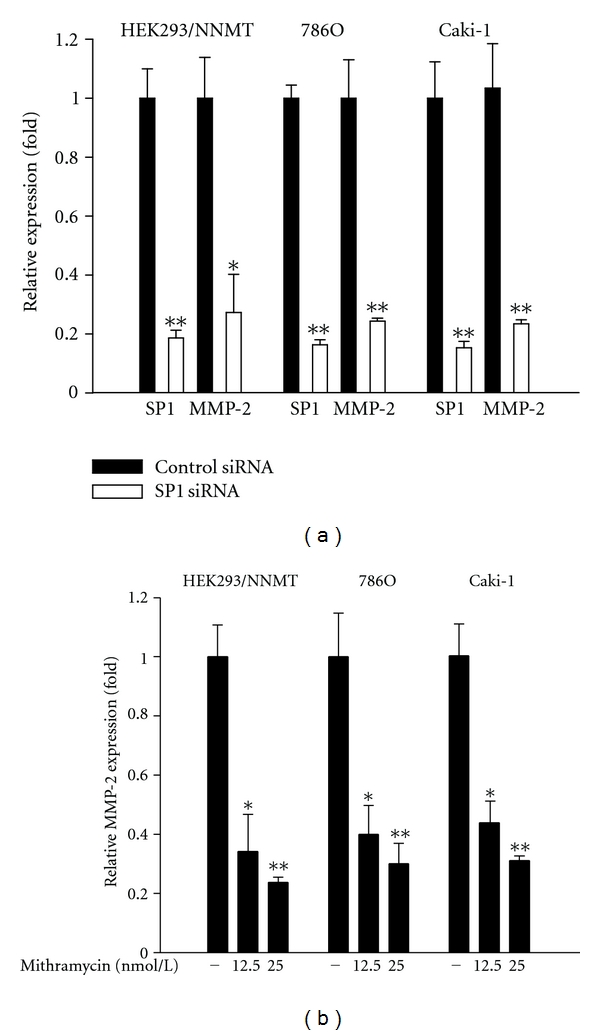
Importance of SP1 in MMP-2 expression induced by NNMT. (a) Effects of SP1 siRNA on the expression of MMP-2 and SP1. Cells were transfected with 40 *μ*mol/L of SP1 siRNA or control siRNA, and Q-PCR was performed to determine the expression levels of NNMT and MMP-2. Data are presented as the mean of triplicate replications. S.D. is indicated by error bars. *P < 0.05; **P < 0.001 versus control cells. (b) Effects of SP1 inhibitor, mithramycin, on the expression of MMP-2. Cells were treated with 12.5 or 25 nmol/L of mithramycin for 18 h, and the expression levels of MMP-2 were examined by Q-PCR. Data are presented as the mean of triplicate replications. S.D. is indicated by error bars. *P < 0.05; **P < 0.001 versus control cells.

To study the crucial region in the MMP-2 promoter responding to NNMT-mediated MMP-2 expression, a 1,716 kb genomic fragment containing the upstream region of MMP-2 gene (WT) and a serial of successive 5′ deletions (D1 to D7 constructs) were cloned into pGL3 luciferase reporter vector [50]. pGL3-MMP-2 promoter plasmid and pGL3 control reporter plasmid were transfected into cells, and the promoter activities were examined using the luciferase reporter assay. As shown in Figure 7(a), a 2-fold increase of the transcriptional activity of the full-length MMP-2 promoter in HEK293/NNMT cells was observed than that in control cells. Additionally, about 4-fold decrease of transcription activities of D6 and D7 mutants, but not D1-D5 mutants, was found as compared with that of the full-length MMP-2 promoter in HEK293/NNMT cells. It indicates that the SP1-binding elements in MMP-2 promoter are required for NNMT-mediated MMP-2 expression. The importance of SP1-binding elements was further examined by using a MMP-2 promoter construct with mutated SP1-binding elements (bp −94 to −64). The results of luciferase reporter assay revealed that the mutation of SP1-binding elements caused a 4-fold reduction of MMP-2 promoter activity in HEK293/NNMT cells (Figure 7(b)). These results suggest that the SP1-binding region of MMP-2 promoter plays a crucial role for NNMT-mediated MMP-2 expression. To study whether SP1 plays a role in NNMT-mediated MMP-2 expression, siRNA specific to SP1 gene was used. As revealed in Figure 8(a), the expression of MMP-2 was markedly reduced by the treatment of SP1 siRNA in HEK293/NNMT, 786O. and Caki-1 cells. Moreover, mithramycin, which has been shown to prevent SP1 binding to gene promoters [51], was used to suppress SP1 transcriptional activity. The results showed that treating with mithramycin apparently suppressed the expression of MMP-2 in HEK293/NNMT, 786O, and Caki-1 cells (Figure 8(b)). It suggests that SP1 is an important factor for MMP-2 expression activated by NNMT. 

**Figure 9:**
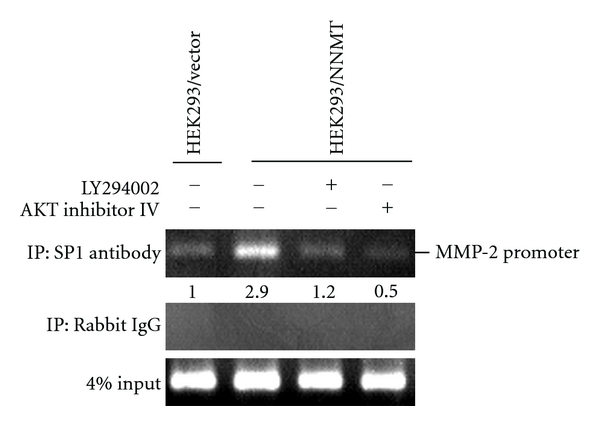
Role of PI3K/AKT pathway in the binding of SP1 to MMP-2 promoter. HEK293/NNMT cells were treated with or without 5 *μ*mol/L of LY294002 or 1 *μ*mol/L of AKT inhibitor IV for 18 h, and the association of SP1 on the SP1 binding elements of the MMP-2 promoter was determined by CHIP assay. Rabbit IgG was used as the negative control and 4% input as positive control.

Furthermore, CHIP assay was carried out to investigate the role of PI3K/AKT pathway in SP1-binding to MMP-2 promoter. The results demonstrated a 2.9-fold increase of SP1 association to the SP1 binding elements of the MMP-2 promoter in HEK293/NNMT cells compared with HEK293/vector cells (Figure 9). Treating with LY294002 or AKT inhibitor IV remarkably reduced the association between SP1 and the MMP-2 promoter in HEK293/NNMT cells (Figure 9). These results strongly implicate that PI3K/AKT/SP1 pathway participates in MMP-2 promoter activation induced by NNMT expression.

## 10. Involvement of MMP-2 in NNMT-Mediated Cell Invasiveness

**Figure 10:**
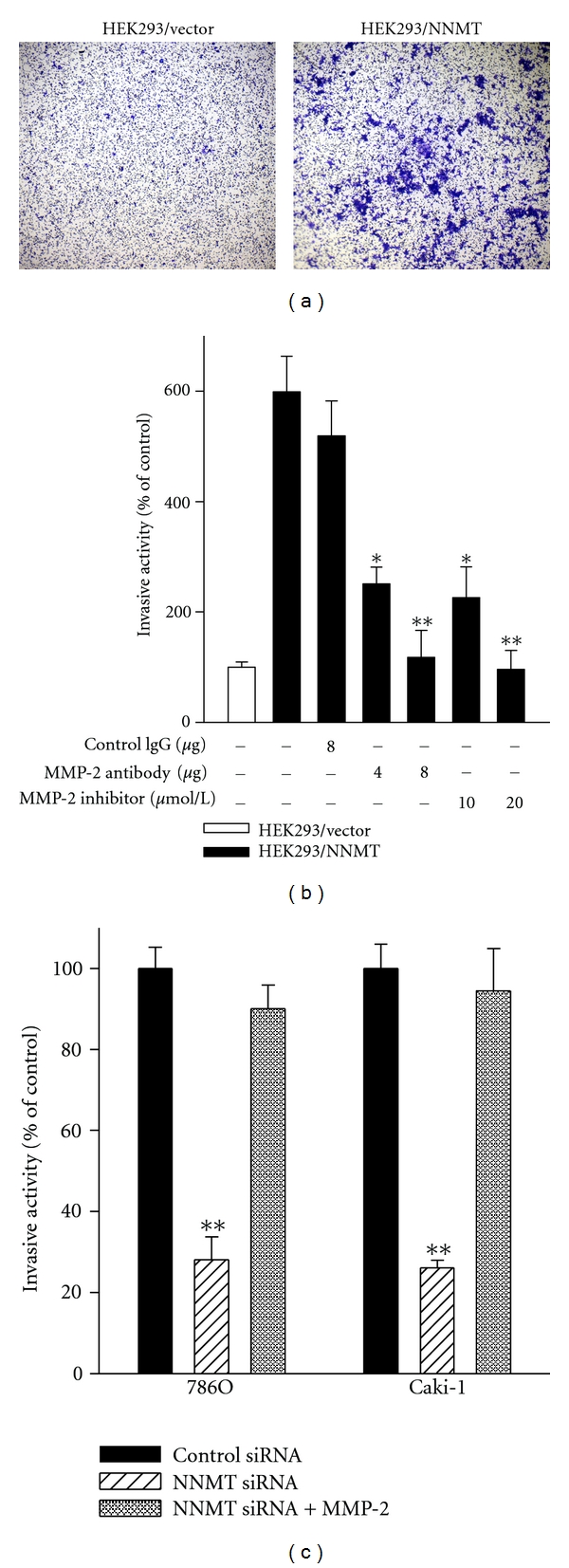
Enhancement of cell invasiveness by NNMT via MMP-2. (a) Matrigel invasion assay was used to analyze the invasive activity. HEK293/vector and HEK293/NNMT cells were seeded into the matrigel-coated chamber and incubated at 37°C for 24 h. (b) Effects of MMP-2-neutralizing antibody or MMP-2 inhibitor OA-Hy on the invasive activity of HEK293/NNMT cells were examined. Data are presented as the mean of triplicate replications. S.D. is indicated by error bars. *P < 0.05; **P < 0.001 versus untreated HEK293/NNMT cells. (c) 786O and Caki-1 cells were transfected with 40 *μ*mol/L of NNMT siRNA for 24 h and then incubated with or without 20 ng of MMP-2 protein in matrigel-coated chamber for another 24 h. Data presented are the mean of triplicate replications. S.D. is indicated by error bars. **P < 0.001 versus control cells.

Whether NNMT-mediated MMP-2 expression causes cell invasion was investigated by matrigel invasion assay. As Figure 10(a) shows, HEK293/NNMT cells exhibited a higher invasive activity than HEK293/vector cells. To examine the role of MMP-2 in NNMT-mediated cell invasiveness, the MMP-2-neutralizing antibody or MMP-2 inhibitor (OA-Hy) were applied to block the activity of MMP-2. The results of matrigel invasion assay indicated that the inhibition of MMP-2 activity significantly suppressed the invasive activity in HEK293/NNMT cells (Figure 10(b)). Additionally, the effects of NNMT siRNA on the invasiveness of ccRCC cell lines were studied. The results showed that treating with NNMT siRNA resulted in about 70% reduction of the invasive activity in 786O and Caki-1 cells (Figure 10(c)). Moreover, NNMT siRNA-suppressed invasiveness was restored by the treatment of MMP-2 active protein (Figure 10(c)). These results indicate that MMP-2 activity is required for the invasiveness of NNMT-overexpressing cells.

## 11. Effects of NNMT shRNA on Tumor Growth and Metastasis of ccRCC Cells in NOD-SCID Mice

**Figure 11:**
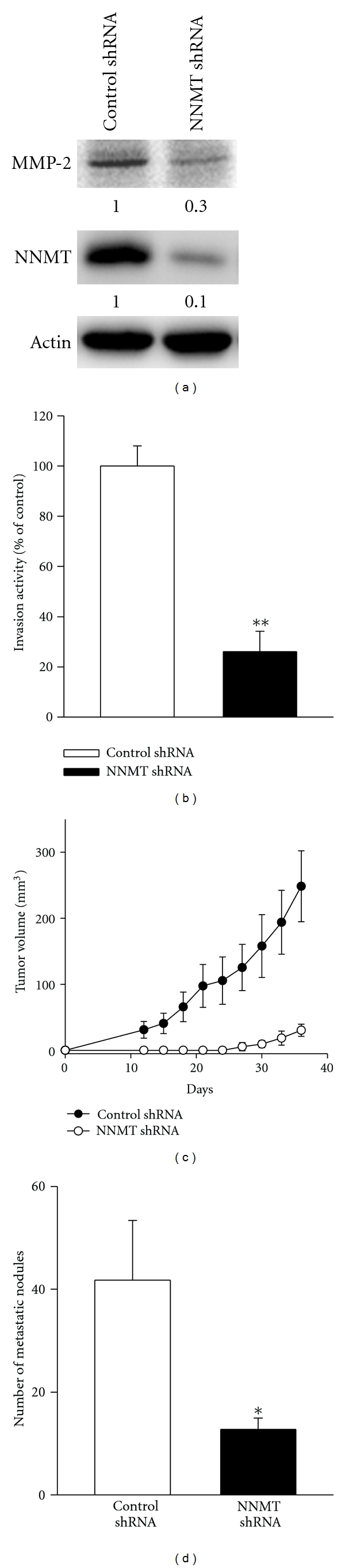
Effects of NNMT shRNA on the growth and lung metastasis of ccRCC cells in NOD-SCID mice. Western blot analysis (a) and matrigel invasion assay (b) were performed to examine NNMT and MMP-2 expression and invasive activities of 786O/NNMT shRNA and 786O/control shRNA cells. Actin was used as loading control. (c) 4 × 10^6^ of 786O/NNMT shRNA or 786O/luciferase shRNA cells were s.c. injected into the hind limb of NOD-SCID mice (*n* = 6). Tumor volume was measured every three days. Data are presented as the mean of triplicate replications. S.D. is indicated by error bars. **P < 0.001 versus control cells. (d) 1 × 10^6^ of cells in 200 *μ*L of serum-free DMEM medium were intravenously injected into the lateral tail vein of NOD-SCID mice (*n* = 6). After 6 weeks of injection, the mice were sacrificed, and the metastatic nodules of lungs were counted. Data are presented as the mean ± SD. *P < 0.05 versus control cells.

Whether the reduced NNMT expression inhibits the tumor growth and metastasis of ccRCC cells was investigated by the experiment of NOD-SCID mice. 786O cells with stably expressing NNMT or luciferase shRNA were generated by using puromycin selection after infected with the lentivirus containing NNMT or luciferase shRNA. As demonstrated by western blot analysis, NNMT shRNA markedly suppressed about 90% of NNMT and 70% of MMP-2 expression in 786O cells (Figure 11(a)). The invasive activity of 786O/NNMT shRNA was reduced to 25% of that of 786O/control shRNA (Figure 11(b)). By subcutaneous injection into the hind limb of NOD-SCID mice, 786O/NNMT shRNA cells developed less tumor mass than 786O/luciferase shRNA cells in NOD-SCID mice (Figure 11(c)). The lung metastatic activity of 786O cells with or without NNMT knockdown was further studied by using intravenous injection into lateral tail vein of NOD-SCID mice. As shown in Figure 11(d), the lung metastasis of 786O cells was significantly suppressed 90% by NNMT shRNA as compared to control shRNA. These results indicate that silenced NNMT expression inhibits tumor growth and lung metastasis of ccRCC cells. 

## 12. Conclusions

Gene expression profiling is one of important methods for studying multifactor diseases and for indentifying the potential biomarkers as the target genes for cancer chemotherapy. We apply the full-length enriched cDNA method to construct the first full-length enriched cDNA libraries of ccRCC and normal kidney tissues. The advantages of present method are (1) the number of upregulated or downregulated genes is obtained more precisely than that of microarray, and (2) the mutations such as point mutation, insertion or deletion, and SNP sites can be identified from the sequencing of full-length enriched cDNA libraries. 

## Figures and Tables

**Table 1:** Differential expression of ccRCC-associated genes in ccRCC.

Unigene number	Gene symbol	Gene name	Location	Average fold change	Percentage (%)
Hs.481022	SFRP2	Secreted frizzled-related protein 2	4q31.3	34.9	50
Hs.511883	ZNF114	Zinc finger protein 114	19q13.33	9.2	32
Hs.164021	CXCL6	chemokine (C-X-C motif) ligand 6 (granulocyte chemotactic protein 2)	4q21	5.0	39
Hs.597524	CDC42	Cell division cycle 42 (GTP binding protein, 25 kDa)	1p36.1	6.0	48
Hs.436367	LAMA3	Laminin, alpha 3	18q11.2	5.7	43
Hs.128453	FRZB	Frizzled-related protein	2qter	6.2	61
Hs.181301	CTSS	Cathepsin S	1q21	8.3	89
Hs.416007	SFRP4	Secreted frizzled-related protein 4	7p14.1	4.3	50
Hs.111779	SPARC	Secreted protein, acidic, cysteine-rich (osteonectin)	5q31.3–q32	4.3	75
Hs.77269	GNAI2	Guanine nucleotide binding protein (G protein), alpha inhibiting activity polypeptide 2	3p21	3.4	50
Hs.480653	ANXA5	Annexin A5	4q28–q32	3.1	36
Hs.168718	AFM	Afamin	4q11–q13	−1020.2	98
Hs.102484	GSTA3	Glutathione S-transferase A3	6p12.1	−72.8	70
Hs.110675	APOE	Apolipoprotein E	19q13.2	−6.4	61
Hs.527971	NES	Nestin	1q23.1	−6.3	48
Hs.9029	KRT23	Keratin 23 (histone deacetylase inducible)	17q21.2	−5.5	50
Hs.54415	CSN3	Casein kappa	4q21.1	−3.7	34
Hs.632294	ZNF38	Zinc finger protein 38	7q22.1	−2.3	41
Hs.389996	CHCHD2	Coiled-coil-helix-coiled-coil-helix domain containing 2	7p11.2	−2.0	36
Hs.458358	TSPYL1	TSPY-like 1	6q22-q23	−2.3	50

**Table 2:** Top 5 functional networks of 383 differentially expressed genes in ccRCC.

1	**ANXA1, ANXA2, ARL6IP1, BRD2, *CTSB, DBI, FBL,* HNRPU, LAMP2, LDHA, *LGMN, MAZ,* MIF, MYC, NAP1L1, NDRG1, NPM1 (includes EG: 4869), PABPC1, *PCBP2, *PGK1, *PRDX1,* PRMT1, *PTBP1, RBMS1, RPL7, RPL22, RPL26, RPL35, RPS3A, RPS4X, *SERINC3, SPP1, SUCLA2, TAGLN2, TMSB4X**	55	35	Protein synthesis, cellular assembly and organization, cancer
2	*14-3-3*, **ALDOC, CCNB1, CCNE1, CD74, *COL1A2,* CTSS, CXCR4, DYRK1A, **E3 RING, ***FBXO18,*** ***H3F3A (includes EG: 3020)***, *Histone h3, * ** Hsp70, Hsp90, HSP90AB1, MAP3K2, *MGMT, *MLL,* NME1,* NOS3, PGAM1, *PRMT5,* PTGES3 (includes EG: 10728), ** *RNA polymerase II, * **SAP18, *SET, SKP1A,* SMARCE1 (includes EG: 6605), *SNRPD2, *ST13, *TCEB2, VHL,* VIM, YWHAE**	40	29	Cancer, cell cycle, cellular compromise

3	***AGT***, *Akt, * **ANXA7, *ATF6,* BCL2, *CA2,* CALM1,** * Calmodulin, * ** CANX, ** *Ck2, * ** CSF1R, DUSP1, EGFR, *EGR1,* ENPEP, GRB10, HSPD1, *KNG1 (includes EG: 3827), *LGALS1, LGALS3, *LTF, *** *Mapk, * ** MET, ** *MHC Class I, P38 MAPK, * ***PIGR, PMP22, *** *Ras, * ***RTN4, *SNX4, SQSTM1, *SRI, *STAT1, *WT1, XBP1 ***	37	28	Cellular development, cellular growth and proliferation, connective tissue development and function

4	*Ap1, * ** ARF1, *ARFIP2, *ATF4, CCND1, CCND3, ** *Creb, Cyclin D, * ** DDX5, EID1,** * Fibrin, * ** ID2, *IGF2, *** * Igfbp, **IGFBP2,*** ** IGFBP3, *KLK5 (includes EG: 25818), *** *Mmp, * **MMP2, MMP7,* MMP24, PCK1, PCK2,* PCNA, ** *PEPCK, * ** PLG, ** *Rb*, ***RFC1, *SERPINA1, SPARC, *SRA1 (includes EG: 10011), TIMP3, *** *Vegf, * **VEGFA, *VTN ***	33	26	Cancer, cellular movement, reproductive system disease

5	**ANGPTL4, *ASS1, ***BNIP3L, ELA2, EREG, **ESM1, **F10, ***GPX4,*** HADHA,** HADHB, **HGF, HIF1A, HNF4A, HSPA5,** IFITM3, *IGF2,* IGFBP3, LIPA, MET, MMP2, NOS3, **NR2F1, ***PHC2, *** **PKM2,** PLAUR, ***POGZ,*** RNF4,** SLC16A4,** SLC2A1, SP1, TERT, **TFPI2, *TRPS1, *** *Vegf, * ** VEGFA**	20	19	Cancer, tumor morphology, cardiovascular system development and function

The score represents the negative log of *P* value that indicates the likelihood of the focus genes in a network being found together due to chance. The number of focus genes reflects number of the differentially expressed genes of ccRCC in a network generated by IPA. (**Bold**): upregulated genes in ccRCC. (***Bold and italic***): downregulated genes in ccRCC. (*Italic*): group or complex. (Standard text): nonfocus genes not included in the 383 differentially expressed genes of ccRCC.

**Table 3:** Expression levels of MYC-target genes in ccRCC and adjacent normal tissues.

Gene name	Normal kidney versus ccRCC^†^ (*P* value^‡^)	Correlation coefficient with MYC expression level of ccRCC tissues (*P* value)^§^
BCL2	−6.58 ± 0.12 versus − 3.84 ± 0.17 (<0.001)	0.45 (0.002)*
CCND1	−4.45 ± 0.14 versus − 2.62 ± 0.18 (<0.001)	0.31 (0.038)*
PCNA	−5.78 ± 0.14 versus − 4.76 ± 0.13 (<0.001)	0.33 (0.038)*
PGK1	−3.40 ± 0.18 versus − 2.70 ± 0.14 (<0.001)	0.42 (0.004)*
VEGFA	−3.93 ± 0.30 versus − 1.23 ± 0.21 (<0.001)	0.36 (0.016)*

^†^Expression levels of MYC-target genes (−ΔCt_GENE-TPT1_  values) measured by Q-PCR in 44 tissues were represented as mean ± SE.

^‡^Significance of difference was determined by paired-samples Student's *t* test.

^§^Correlation between the expression levels of MYC and MYC-target genes in ccRCC tissues was determined by *Pearson* correlation method.

*P < 0.05 was considered statistically significant.
